# Prevalence and impact of baseline resistance-associated substitutions on the efficacy of ledipasvir/sofosbuvir or simeprevir/sofosbuvir against GT1 HCV infection

**DOI:** 10.1038/s41598-018-21303-2

**Published:** 2018-02-16

**Authors:** Gary P. Wang, Norah Terrault, Jacqueline D. Reeves, Lin Liu, Eric Li, Lisa Zhao, Joseph K. Lim, Giuseppe Morelli, Alexander Kuo, Josh Levitsky, Kenneth E. Sherman, Lynn M. Frazier, Ananthakrishnan Ramani, Joy Peter, Lucy Akuskevich, Michael W. Fried, David R. Nelson

**Affiliations:** 1 University of Florida, Gainesville, USA; 2 North Florida/South Georgia Veterans Health System, Gainesville, USA; 3 University of California, San Francisco, San Francisco, USA; 4 Monogram Biosciences, South San Francisco, USA; 5 Yale University School of Medicine, New Haven, USA; 6 Virginia Mason Medical Center, Seattle, USA; 7 Northwestern University Feinberg School of Medicine, Chicago, USA; 8 University of Cincinnati, Cincinnati, USA; 9 Liver Wellness Center, Little Rock, USA; 10 Columbia Memorial Hospital (Mountainview Medical Center), Hudson, USA; 11 University of North Carolina, Chapel Hill, United States

## Abstract

Baseline resistance-associated substitutions (RASs) have variable impacts in clinical trials but their prevalence and impact in real-world patients remains unclear. We performed baseline resistance testing using a commercial assay (10% cutoff) for 486 patients treated with LDV/SOF or SMV/SOF, with or without ribavirin, in the multi-center, observational HCV-TARGET cohort. Linkage of RASs was evaluated in selected samples using a novel quantitative single variant sequencing assay. Our results showed that the prevalence of NS3, NS5A, NS5B RASs was 45%, 13%, and 8%, respectively, and 10% of patients harbored RASs in 2 or more drug classes. Baseline LDV RASs in GT1a, TE, and cirrhosis LDV/SOF subgroup was associated with 2–4% lower SVR12 rates. SMV RASs was associated with lower SVR12 rates in GT1a, treatment-experienced, cirrhotics SMV/SOF subgroup. Pooled analysis of all patients with baseline RASs revealed that SVR12 was 100% (19/19) in patients treated for longer than 98 days but was 87% (81/93) in patients treated for shorter than 98 days. These results demonstrate that RASs prevalence and their impact in real world practice are in general agreement with registration trials, and suggest that longer treatment duration may overcome the negative impact of baseline RASs on SVR12 rates in clinical practice.

## Introduction

According to the latest WHO estimates, approximately 1% of the world’s population is infected with hepatitis C virus (HCV), corresponding to 71 million people worldwide^1,2^. Chronic hepatitis C is the most common cause of liver cirrhosis and is the leading indication of liver transplantation due to cirrhosis and hepatocellular carcinoma. Chronic HCV is responsible for 12,000 deaths annually, and the morbidity and mortality associated with HCV infection will continue to increase over the next few decades^3–5^.

Several direct acting antivirals (DAAs) have been developed with multiple options now to treat HCV genotype 1 (GT1) infection, the predominant genotype in the U.S. With the development of DAAs, multiple regions of HCV (NS3, NS5A, and NS5B) are major targets for pharmacologic intervention leading to highly potent treatment combinations with sustained virologic response (SVR) rates well above 90%^6–12^. Although recently approved glecaprevir-pibrentasvir^13^ and sofosbuvir-velpatasvir-voxilaprevir^9^ with pangenotypic activity and a high barrier to resistance may be used increasingly and may also fill an important role as pangenotypic regimens for patients who previously failed DAA therapy, up to 5–10% of treated patients still fail some of the current recommended first-line DAA regimens, which leads to the selection of resistant variants, making regimen selection a major consideration for providers and patients.

The nomenclature for resistant HCV has varied in recent years, including resistance-associated substitutions (RAS), resistance-associated variants (RAV), and resistance-associated polymorphisms (RAP). HCV replicates at high rates allowing for rapid response to selective immune or drug induced pressure which may select for resistant variants *in vivo*^14^. Viral resistance-associated substitutions (RASs) have also been observed in patients naïve to HCV treatment (i.e. pre-existing) and RASs can develop in the setting of DAA therapy. Clinical trials have demonstrated that baseline resistance can play a role in HCV treatment response. For example, in the COSMOS study combining simeprevir (protease inhibitor) with sofosbuvir (SOF, NS5B nucleoside inhibitor), 58 genotype 1a patients had the Q80K substitution in the NS3 region at baseline, 7 of those failed to achieve SVR12^11^. In a combined analysis of baseline samples from Phase 2 and Phase 3 Gilead-sponsored HCV studies, baseline NS5A resistance mutations were present in 13% of 3,507 samples. Among patients treated with ledipasvir (LDV) plus SOF, lower SVR12 rates (88.2%; 105 of 119) were observed in treatment experienced patients with baseline NS5A RASs as compared to those without NS5A RASs (SVR12 of 97.7%, 646/661)^15^. In the C-EDGE trial combining grazoprevir (NS3 protease inhibitor) with elbasvir (NS5A inhibitor), 58% (11/19) of GT1a patients with baseline RASs achieved SVR12, whereas 99% (133/135) of patients without baseline RASs achieved SVR12^12^. If high fold-change RASs (i.e. >5-fold increase in EC50) are considered, only 22% (2/9) of patients achieved SVR12. This large impact of baseline NS5A RASs on treatment outcome has led the FDA to recommend baseline NS5A resistance testing in GT1a patients prior to grazoprevir/elbasvir therapy.

While the presence of baseline RASs has impacted viral outcomes in small numbers of patients in tightly controlled clinical trials, the prevalence and the range of baseline RASs and the extent to which they impact the efficacy of DAAs in diverse, real-world populations, including certain sub-populations (e.g. treatment-experienced, cirrhosis, liver transplant recipients) in usual clinical practice remains unclear. Real-world patients receiving treatment outside of highly selected clinical trial populations have diverse baseline characteristics and many have prior exposure to one or more DAAs. The commercial availability of resistance testing is limited and clinicians often do not test patients for baseline resistance prior to initiating therapy. Moving into an era where DAA combinations will be heavily used in real-world populations, viral factors such as HCV genotype, genome targets, and drug resistant variants must be balanced with host factors including cirrhosis and prior treatment response in order to select the optimal regimen for each individual patient.

HCV-TARGET is an IRB approved, carefully maintained research registry of sequential patients treated for chronic HCV within academic and community real-world practices. The registry was designed to rapidly inform strategies for better management of populations underrepresented in clinical trials, identify and remediate educational gaps relative to treatment guidelines and adverse event management in order to optimize rates of SVR, as well as to serve as the core resource for important collaborative translational studies utilizing biospecimens and clinical data from diverse patient populations. Serum samples are collected within HCV-TARGET for research towards the understanding of HCV pathogenesis and treatment. To date, over 10,000 patients have been enrolled into the study with over 8,600 being enrolled since the FDA approval of simeprevir and sofosbuvir in late 2013. Here, we describe the prevalence of NS3, NS5A, and NS5B RASs in a real-world cohort of ~500 patients with HCV genotype 1 infection whose baseline samples were banked prior to DAA therapy, and report the impact of baseline RASs on the effectiveness of ledipasvir/sofosbuvir (LDV/SOF) ± ribavirin (RBV) and simeprevir/sofosbuvir (SMV/SOF) ± RBV.

## Methods

### Study population

A subset of HCV-TARGET patients consented to serum collection and specimen storage in the “HCV-TARGET: Biorepository Specimen Bank” prior to initiating HCV therapy. All patients included in this study (n = 494) have provided informed consent to study participation. Subjects were included for the present study if (1) patient in the HCV-TARGET cohort consented to stored serum for future research, (2) pre-treatment serum sample was collected and stored at the HCV-TARGET biorepository housed at the University of Florida (UF), (3) HCV genotype 1 infection, and (4) patient was treated with ledipasvir/sofosbuvir (LDV/SOF) ± ribavirin (RBV) or simeprevir/sofosbuvir (SMV/SOF) ± RBV. Baseline and treatment clinical data for the subjects were extracted from REDCap database for analysis. The present study was approved by University of Florida Institutional Review Board, and all experiments were performed in accordance with relevant guidelines and regulations.

### Determination of resistance-associated substitutions (RASs)

Resistance testing was performed on baseline samples by Monogram Biosciences (South San Francisco, CA) using their commercial assay. The assay reports the population sequence derived from Illumina MiSeq data using a 10% variant reporting threshold. 494 genotype 1 patients samples were deep sequenced and evaluated against a reference sequence (H77 for GT1a and Con-1 for GT1b) for NS3 protease inhibitor (PI), NS5A inhibitor, and NS5B nucleotide (NI) and non-nucleotide (NNI) inhibitor RASs. HCV genotype subtype 1a and 1b were verified along with the presence of Q80K polymorphism in genotype 1a patients.

### Analysis of RASs

Baseline clinical and demographic data, HCV treatment regimen, and viral outcomes were paired with RAS testing results for descriptive analyses. Percentage of patients in different patient subgroups with baseline NS3 protease inhibitor, NS5A inhibitor and NS5B nucleotide and non-nucleotide inhibitor RASs were analyzed using descriptive statistics and their proportions compared using Fisher’s exact test. For analysis of prevalence of baseline RASs, NS3 RASs were defined as the following substitutions at the following positions: V36M/A/G/I/L, T54A/S, V55A/I, Q80K/R, S122A/G/I/R/T, R155K/Q, A156T, D168A/E/F/H/I/T/V/Y, and I/V170T. NS5A RASs were defined as M28A/T/V (1a), L28M (1b), Q30E/H/L/R (1a), L31I/M/V, H58D (1a), and Y93C/H/N/S. NS5B RASs were defined as L159F, S282R/T, C316F/H/N, L320F/I/V, V321A/I, M414T/I, Y448H, A553T, G554S, S556G, and D559G/N. For analysis of the impact of baseline RASs on treatment response, a subset of positions and their substitutions shown above were included in the analysis: NS3: 80, 122, 155, 156, 168, and 170; NS5A: 28, 30, 31, 58, and 93; NS5B: 159, 282, 316, 320 and 321 (Supplementary Table S1).

### Quantitative resistance analysis and mutation linkage

HCV NS3, NS5A or NS5B gene segments were amplified and sequenced from extracted RNA using the SVS methods described previously^16^. Briefly, a random 12-nt sequence (i.e. tag) was incorporated into the 5’ end of the reverse-transcription (RT) primer to tag individual RNA templates during RT reaction, and this random sequence tag was flanked by a primer binding sequence on the 5′ end, which served as a binding site for subsequent PCR reactions. Following RT, excess RT primers were removed using a Macherey-Nagel DNA purification column using a modified protocol following manufacturer’s instruction (Macherey-Nagel, Bethlehem, PA, USA). The cDNA was PCR amplified using barcoded primers, and tailed with Illumina adaptor sequences required for deep sequencing. PCR conditions were: initial denaturation at 94 °C for 2 min followed by 30 cycles of 94 °C for 20 s, 56 °C for 20 s, and 68 °C for 1 min, an extra 5 min at 68 °C was added at the end of amplification. Primers were designed based on a curated set of 390 subtype 1a or 453 subtype 1b full-length human HCV sequences from GenBank that represent HCV genomes from popular circulating strains^17^. Final PCR products were purified using a gel extraction kit (Macherey-Nagel, Bethlehem, PA, USA), quantified using a Qubit kit (Life technologies), and pooled with equimolar concentrations. The concentration of the final DNA pool was quantified by real-time PCR using a SYBR Green qPCR kit (KAPA Biosystems). The DNA library was then prepared and sequenced on a MiSeq sequencer (Illumina, San Diego, CA) following manufacturer’s instructions. A Q30 filter was used to select for high quality reads, generating a total of 5.62 Gigabases of nucleotides. Insertions and deletions were found to be minimal in our sequence dataset.

### Bioinformatics analysis of HCV resistance variants

Illumina paired-end sequencing data were de-multiplexed into individual samples according to unique combinations of forward and reverse barcodes, followed by additional filtering criteria to select for high-quality reads. Each paired-end read was joined using FLASh (http://ccb.jhu.edu/software/FLASH/) with a minimum of 10 base overlap. For each sample, joined reads were grouped by unique 12-nt sequence tags introduced during the RT reaction. To determine the authentic sequence of each initial cDNA template and correct for PCR amplification and sequencing errors, a consensus sequence was determined for each unique sequence tag based on an alignment of at least three reads using MAFFT (http://mafft.cbrc.jp/alignment/software/). Those with fewer than 3 reads per unique tag were discarded. Consensus sequences were then aligned against HCV-H77 (NC004102) or HCV-CON1 (AJ238799) reference sequence using a 36 CPU Amazon Web Services EC2 Ubuntu Linux (version 14.04) instance (https://aws.amazon.com/ec2), and visualized using BioEdit (version 7.2.5.0; http://www.mbio.ncsu.edu/BioEdit/bioedit.html).

Translation of codons, calculation of proportions of RASs, and linkage analysis of RASs were carried out using custom R scripts (https://www.r-project.org/) with the BioStrings package (http://bioconductor.org/packages/release/bioc/html/Biostrings.html). Amino acid substitutions at each position were identified by comparing translated consensus reads against HCV H77 or CON-1, and the proportions of wild-type and RASs at each position were calculated. Linkage between RASs was determined by calculating the proportions of variants carrying single, double or multiple RAS(s) compared to the reference sequence.

## Results

### Study population

**Figure 1:**
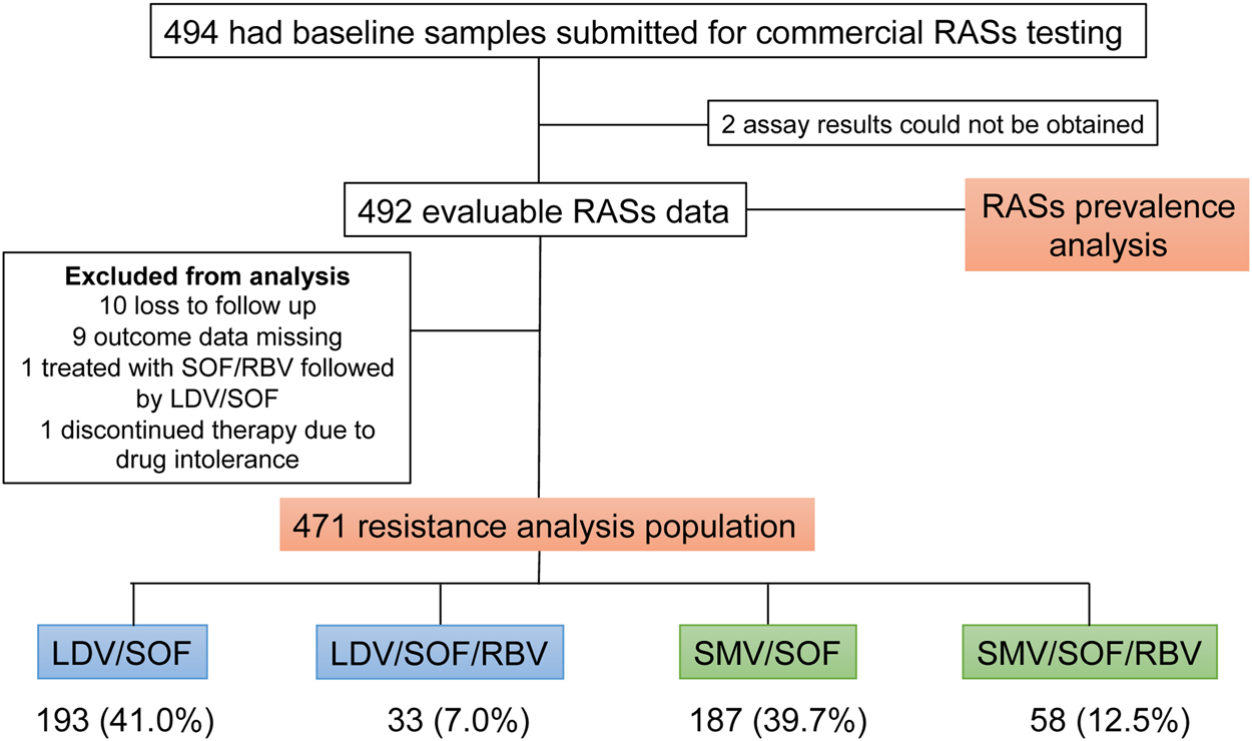
Selection of baseline samples from patients enrolled in HCV-TARGET for the study. Baseline samples from 492 patients with evaluable RASs were used to determine the overall prevalence of baseline RASs. Among them, 472 patients with clinical outcome data were selected and the impact of baseline RASs on SVR12 for each of the four treatment regimens was analyzed.

From the “HCV-TARGET: Biorepository Specimen Bank”, we identified 494 patients who met our inclusion criteria. Pretreatment samples of these subjects were submitted to Monogram Biosciences for sequencing and RASs determination. Of the 494 samples, 492 had evaluable RASs sequence dataset, from which the prevalence of NS3, NS5A, and NS5B RASs was determined (Fig. 1). To examine the impact of baseline RASs on the effectiveness of LDV/SOF ± ribavirin (RBV) or SMV/SOF ± RBV, we excluded 21 of 492 patients who were lost to follow up, had missing outcome data, were treated with additional regimens, or discontinued therapy prematurely due to drug intolerance, generating a dataset of 471 subjects with evaluable treatment outcomes (Fig. 1).

### Prevalence of RASs in routine clinical practice

Demographics and baseline characteristics of 492 subjects with evaluable RASs dataset for analysis of RASs prevalence are shown in Table 1. Overall, the median age in this cohort was 60 years. The study population was predominantly white (70.7%) and male (62.6%), with 75.6% GT1a and 24.4% GT1b. Approximately half of the cohort was treatment-experienced (TE, n = 270; 54.9%), the majority of whom (185 of 270, 68.5%) had prior treatment with pegylated interferon plus ribavirin. Overall, 63 (12.8%) patients were DAA-experienced. These include 50 patients with prior exposure to triple therapy (pegylated interferon, ribavirin and either boceprevir or telaprevir), 5 patients with simeprevir and sofosbuvir, 4 patients with sofosbuvir, 1 with declatasvir, 1 with simeprevir, and 1 with an unknown DAA. A large proportion of patients had cirrhosis (52%) or were recipients of liver transplantation (17.7%).

**Table 1:** Demographics and Baseline Characteristics of 492 subjects with evaluable RASs dataset. Liver disease stage (cirrhosis or no cirrhosis) was defined at the time of enrollment by biopsy and/or or a combination of clinical, laboratory, histologic, and imaging criteria features as defined previously^31,35^. The LDV/SOF group included one patient who was treated with SOF/RBV followed by LDV/SOF. Only 36% of all patients had available IL28B data and thus were not included in the comparison.

		LDV/SOF	LDV/SOF + RBV	SMV/SOF	SMV/SOF + RBV	Total
		n = 208	n = 34	n = 190	n = 60	n = 492
Median age (quartile)	Age	61 (57, 65)	60 (54, 64)	60 (56, 65)	60 (54, 63)	60 (56, 65)
Gender, n (%)	Male	124 (59.6)	24 (70.6)	124 (65.3)	36 (60.0)	308 (62.6)
Race, n (%)	White	138 (66.4)	19 (55.9)	143 (75.3)	48 (80.0)	348 (70.7)
	Black	38 (18.3)	2 (5.9)	16 (8.4)	7 (11.7)	63 (12.8)
	Other	31 (16.0)	13 (38.2)	31 (16.3)	5 (8.3)	81 (16.5)
Prior treatment status, n (%)	TN	106 (51.0)	11 (32.4)	83 (43.7)	22 (36.7)	222 (45.1)
	TE	102 (49.0)	23 (67.7)	107 (56.3)	38 (63.3)	270 (54.9)
	DAA hx	23 (11.1)	14 (41.2)	15 (7.9)	11 (18.3)	63 (12.8%)
Cirrhosis status, n (%)	Cirrhosis	85 (40.9)	21 (61.8)	109 (57.4)	41 (68.3)	256 (52.0)
Liver transplant status, n (%)	Transplant	19 (9.1)	19 (54.3)	38 (19.9)	11 (18.0)	87 (17.7)
Genotype	GT 1a	162 (77.9)	27 (79.4)	133 (70.0)	50 (83.3)	372 (75.6)
	GT 1b	46 (22.1)	7 (20.6)	57 (30.0)	10 (16.7)	120 (24.4)
Mean HCV RNA, log IU/mL	Viral load	6.6	6.7	6.6	6.8	6.6

Of the 492 patients with evaluable RASs data, 482 (98%) had RASs data available for all 3 gene targets (NS3, NS5A, and NS5B). A total of 486 samples had NS3 RASs data, 490 with NS5A data, and 486 with NS5B data. Discordance in genotype 1 subtype between the clinical data submitted by clinical sites and the sequence data generated by the Monogram resistance assay was identified in 11 subjects (2.2%; 7 were submitted as GT1b but were later determined as GT1a at Monogram, and 4 were submitted as GT1a but were later determined as GT1b). For consistency, the subtype data reported by the Monogram assay was used for all subsequent analysis.

**Figure 2:**
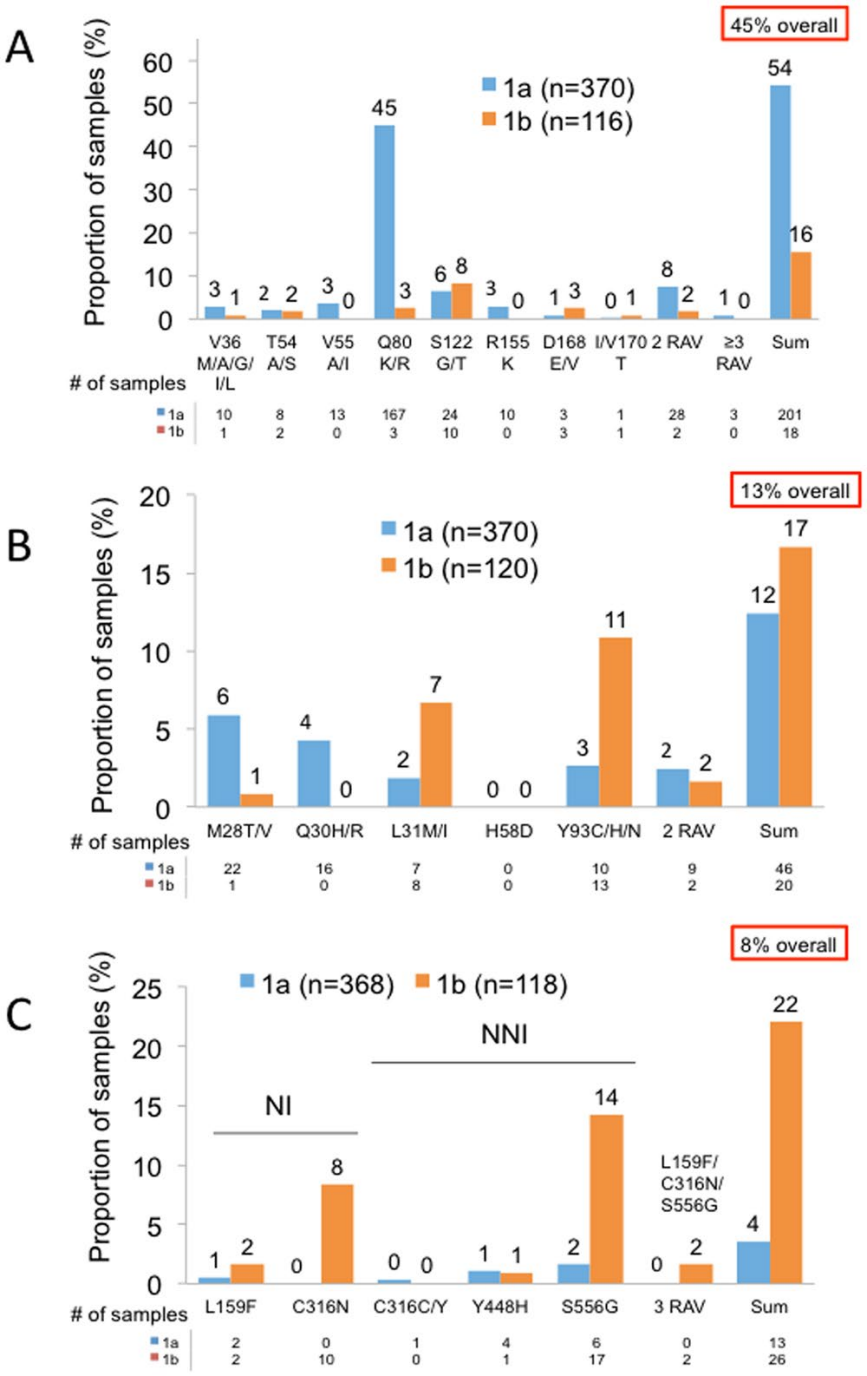
Prevalence of RASs in (**A**) NS3, (**B**) NS5A, and (**C**) NS5B in 492 patients prior to initiating HCV therapy. Specific RASs are indicated on the x-axis. The proportion of samples with substitutions indicated, as determined by the commercial Monogram assay, is shown on the y-axis. Samples for GT1a and G1b are shown in blue and orange, respectively. Substitutions at positions NS3 A156, NS5A H58D, and NS5B S282, L320, or V321 were not detected in any of the samples analyzed. The proportion of samples with 2 or more RASs, and the overall proportions of samples with RASs are shown on the right.

To determine the prevalence of NS3, NS5A and NS5B RASs in patients prior to initiating HCV therapy, we defined the presence or absence of specific RASs using a set of pre-defined amino acid substitutions (Supplementary Table S1). For NS3, Q80K/R substitution was common in GT1a (45%; 44% Q80K and 1% Q80R) but uncommon in GT1b (2.6%) (Fig. 2A). RASs at other pre-defined sites were generally uncommon, and RASs at position A156 were not observed in any of the 486 samples. Overall, 45% of patients (54% of GT1a and 16% of GT1b) harbored NS3 RASs at at least 1 position, and 8% of GT1a and 2% of GT1b harbored RASs in 2 or more positions.

The overall prevalence of RASs was lower in NS5A compared to NS3 (13% vs 45%) (Fig. 2A and B). GT1a NS5A RASs were generally uncommon, ranging from 2% (L31M/I) to 6% (M28T/V). Y93C/H/N substitution, which confers a high level resistance to most NS5A inhibitors, were uncommon (3%). In contrast, Y93C/H/N and L31M/I substitutions were more common in GT1b (11% and 7%, respectively), but RASs at other pre-defined sites were uncommon. Overall, 46 GT1a (12%) and 20 GT1b (17%) patients harbored RASs in at least 1 position (Fig. 2B), including 11 (2%) who harbored RASs at 2 different positions.

The overall prevalence of NS5B RASs was 8% (3.6% in GT1a compared to 22% in GT1b) (Fig. 2C). GT1a NS5B RASs were uncommon. In contrast, C316N and S556G substitutions in GT1b were common (8% and 14%, respectively). Interestingly, two GT1b subjects harbored 3 different RASs (L159F/C316N/S556G), but no GT1a subjects harbored more than 1 RAS. RASs at positions S282, L320, and V321 were not detected in any of the 486 samples.

We next asked to what extent the prevalence of RASs differed according to different host and viral characteristics. The overall prevalence of RASs was generally comparable between cirrhotics and non-cirrhotics, treatment-naïve and treatment-experienced (who were predominantly DAA-naïve), or liver transplant status (Table 2). However, there were small differences according to subpopulations. For example, NS3 RASs were more prevalent in GT1a (driven predominantly by the high frequency of Q80K/R substitutions in GT1a), cirrhotics, DAA-experienced, and liver transplant population. NS5A RASs were more common in GT1b, treatment-experienced, DAA-experienced, and non-transplant populations. For NS5B, the prevalence of RASs was higher in GT1b. Overall, 10% of patients harbored RASs in 2 or more drug classes. Among them, NS3/NS5A RASs combination was most common (65%), followed by NS3/NS5B (23%) and NS5A/NS5B (12%).

**Table 2:** Frequency of Baseline RASs in Specific Sub-Populations. The numbers represent the proportion of patients (95% CI) in a given sub-population (e.g. GT1a) who harbored RASs in the gene targets indicated (e.g. NS3), or in two or more gene classes. RASs were determined using the Monogram commercial assay.

	NS3	NS5A	NS5B	≥2 classes
Genotype 1a	54.3 (49.2–59.3)	12.4 (9.4–16.2)	3.5 (2.1–6.0)	9.7 (7.1–13.1)
Genotype 1b	15.5 (10.1–23.2)	16.7 (11.1–24.4)	22.0 (15.5–30.3)	15.0 (9.7–22.5)
No cirrhosis	39.7 (33.6–46.1)	13.6 (9.8–18.5)	8.6 (5.6–12.9)	9.3 (6.2–13.7)
Cirrhosis	50.0 (43.9–56.1)	13.4 (9.7–18.1)	7.5 (4.9–11.4)	12.5 (9–17.1)
TN	43.4 (37.1–49.9)	11.4 (7.9–16.2)	7.9 (5.1–12.2)	8.8 (5.8–13.2)
TE	46.5 (40.6–52.6)	15.3 (11.4–20.1)	8.1 (5.4–12.0)	12.9 (9.4–17.5)
DAA	60.0 (44.6–73.6)	15.0 (7.1–29.1)	0 (0.0–8.8)	10.0 (4.0–23.1)
No Transplant	43.1 (38.3–48.0)	15.1 (12.0–19.0)	7.5 (5.3–10.5)	11.1 (8.4–14.6)
Transplant	53.5 (43.0–63.7)	5.8 (2.5–12.9)	10.6 (5.7–18.9)	10.3 (5.5–18.5)

### LDV RASs and efficacy of LDV/SOF ± RBV in routine clinical practice

To evaluate the impact of baseline RASs on the effectiveness of LDV/SOF ± RBV or SMV/SOF ± RBV, we analyzed a subset of key RASs (Supplementary Table S1) that were relevant for the DAA regimen that the patients received in clinical practice. The majority of this subset received LDV/SOF or SMV/SOF without RBV (41.0% and 39.7%, respectively), and the remaining patients received LDV/SOF or SMV/SOF with RBV (7.0% and 12.3%, respectively).

**Figure 3:**
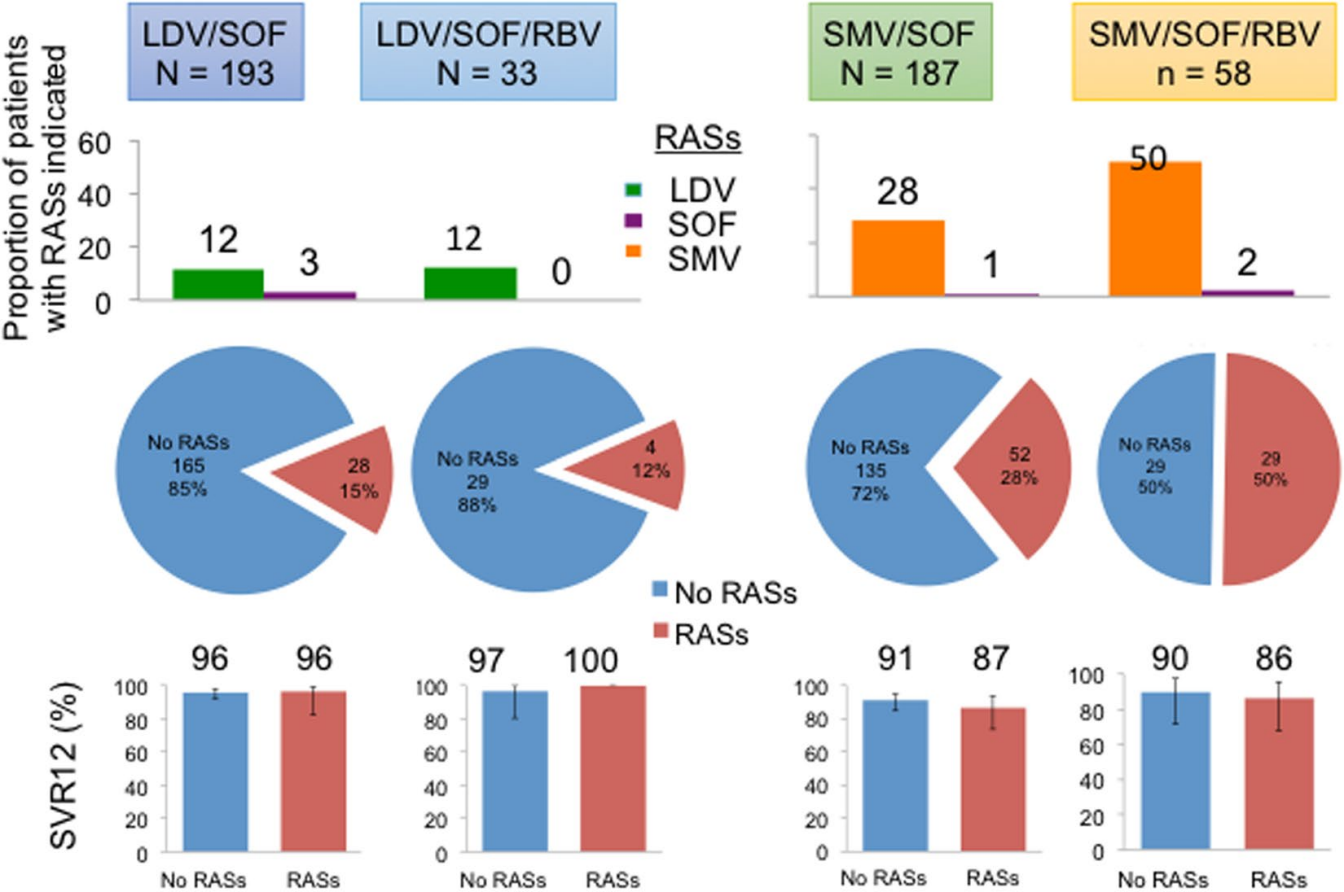
Efficacy of DAA regimens with baseline RASs. (Top) The proportions of patients with LDV, SOF, or SMV-specific RASs are shown in green, purple and orange bars, respectively. (Middle) The proportions of patients with RASs relevant for the treatment regimens are indicated in red. For example, for the LDV/SOF cohort, total number of patients with baseline RASs included patients with LDV and/or SOF RASs. (Bottom) The proportions of patients with or without baseline RASs who achieved SVR12 are shown in red and blue bars, respectively.

**Figure 4:**
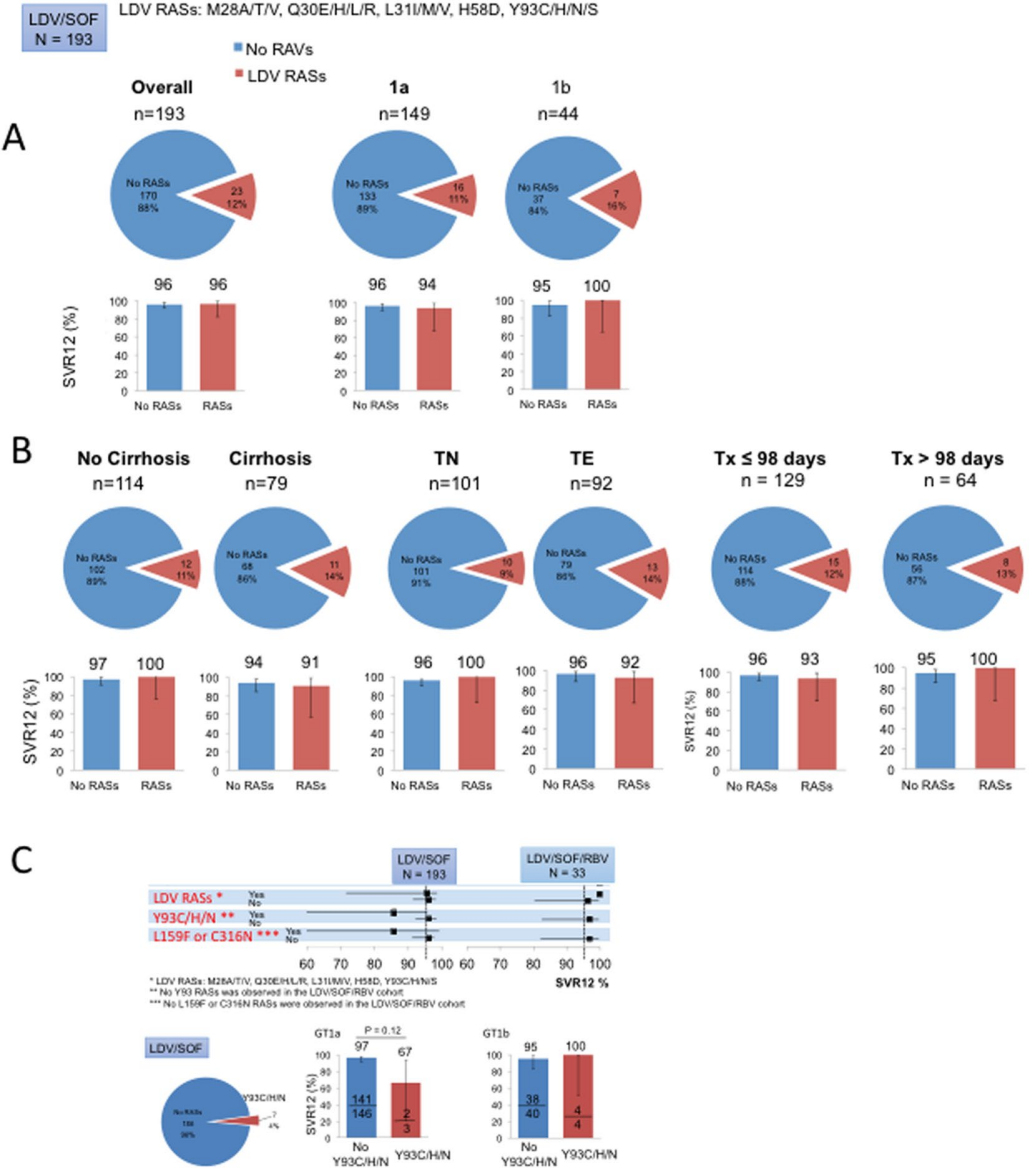
Efficacy of LDV/SOF with baseline LDV RASs in different patient subpopulations. (**A**) and (**B**). Top: Proportions of patients with baseline LDV RASs are shown in red. Bottom: Proportions of patients with or without baseline LDV RASs are shown in red and blue bars, respectively. (**C**) Top: SVR12 rates of patients with or without specific RASs indicated on the left are shown with 95% CI. Bottom left: Proportion of patients with Y93C/H/N RASs. Bottom right: Proportions of patients with or without Y93C/H/N RASs who achieved SVR12 are shown in red and blue, respectively. LDV RASs analyzed were M28A/T/V, Q30E/H/L/R, L31I/M/V, H58D, Y93C/H/N/S. No Y93 RASs was observed in the LDV/SOF/RBV cohort. No L159F or C316N RASs were observed in the LDV/SOF/RBV cohort.

In the LDV/SOF cohort (n = 193), 22 patients (12%) harbored LDV-associated RASs, 5 (2.6%) had SOF-associated RASs, and 1 patient had both LDV and SOF-associated RASs prior to HCV therapy (Fig. 3). 22 of 23 patients (96%) with LDV-associated RASs (positions 28, 30, 31, 58 and 93) achieved SVR12, and 163 of 170 subjects (96%) without LDV RASs (Fig. 4A). The presence of baseline LDV RASs was associated with 2% (p = 0.50), 3% (p = 0.54), and 4% (p = 0.46) lower SVR12 rates in GT1a, cirrhotic, or treatment-experienced patients, respectively (Fig. 4B). Baseline LDV RASs was associated with 3% lower SVR12 rates in patients who received ≤98 days of therapy. In contrast, all 8 patients with baseline LDV RASs who were treated for >98 days achieved SVR12 (Fig. 4B). Of the 193 patients in the LDV/SOF cohort, 9 patients were treated for 8 weeks and 8 of 9 (89%) achieved SVR12. All 9 patients were treatment-naïve without cirrhosis and had HCV RNA <6 million IU/mL. However, none had baseline NS5A or NS5B RASs. The single patient who relapsed was white, GT1b, and HIV-uninfected with a baseline HCV RNA of 3 million IU/mL. Among the 7 patients with baseline Y93 RASs (3 GT1a and 4 GT1b), only one patient (GT1a) failed to achieve SVR12 (Fig. 4C). This patient harbored dual RASs (Q30H/N/S + Y93H; detailed analysis of this viral quasispecies is described below), was treatment-experienced (pegylated interferon plus ribavirin), DAA-naïve, GT1a with cirrhosis, and developed viral breakthrough during 12 weeks of therapy. For NS5B RASs, 179 of 186 patients (95%) without baseline NS5B L159F or C316N substitutions and 6 of 7 patients (86%) with baseline L159F or C316N achieved SVR12. The single GT1b patient with NS5B RAS who failed to achieve SVR12 harbored C316N RAS, was treatment-naïve with no cirrhosis, and was treated for 12 weeks of LDV/SOF. In the LDV/SOF + RBV cohort (n = 33), LDV-associated RASs had no impact on SVR12, and no Y93C/H/N, L159F, or C316N substitutions were observed at baseline. Baseline NS3 RASs had no impact on SVR12 rates in the LDV/SOF ± RBV cohorts (Supplmentary Figure S4).

### SMV RASs and efficacy of SMV/SOF ± RBV in routine clinical practice

**Figure 5:**
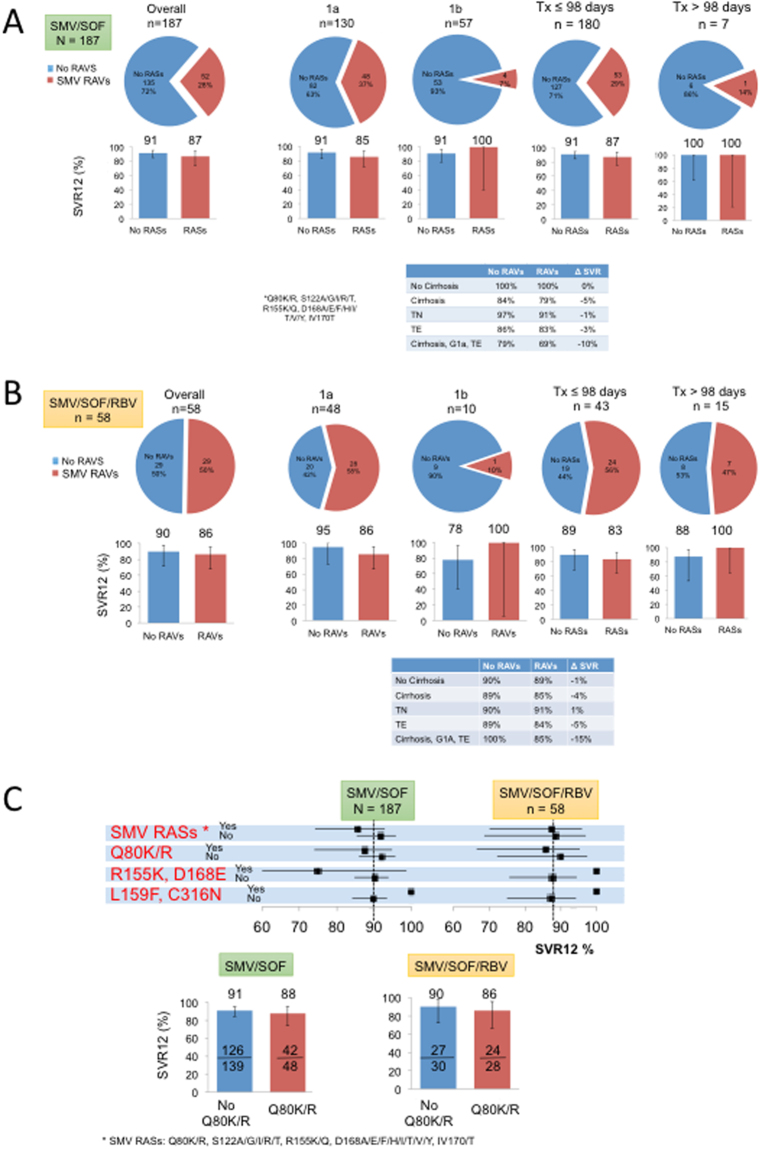
Efficacy of SMV/SOF with baseline SMV RASs in all patients, by genotype 1 subtype, and by patient subpopulations. (**A**) and (**B**). Top: Proportions of patients with baseline SMV RASs are shown in red. Bottom: Proportions of patients with or without baseline SMV RASs are shown in red and blue bars, respectively. (**A**) Patients treated with SMV/SOF, (**B**) Patients treated SMV/SOF//RBV. (**C**) Top: SVR12 rates of patients with or without specific RASs indicated on the left are shown with 95% CI. Bottom left: Proportion of patients with Q80K/R. Bottom right: Proportions of patients with or without Y93C/H/N RASs who achieved SVR12 are shown in red and blue, respectively. SMV RASs analyzed were Q80K/R, S122A/G/I/R/T, R155K/Q, D168A/E/F/H/I/T/V/Y, IV170/T.

Baseline SMV-associated RASs were common in the SMV/SOF (28%, 52 of 187) and SMV/SOF + RBV (50%, 29 of 58) cohorts (Fig. 3). As in LDV/SOF cohorts, SOF-associated RASs were uncommon. In the SMV/SOF cohort (n = 187), 45 of 52 patients (87%) with baseline SMV RASs achieved SVR12, whereas 123 of 135 patients (91%) without SMV RASs achieved SVR12 (Fig. 5A). In GT1a, the presence of SMV RASs was associated with a small reduction in SVR12 (85% vs 91%; p = 0.38). In contrast, SMV RASs had no impact on SVR12 in GT1b. Baseline SMV RASs was associated with 4% lower SVR12 rates in patients who received ≤98 days of therapy. All 7 patients treated for >98 days achieved SVR12 regardless of baseline SMV RASs (Fig. 5A). In patients with cirrhosis or who were treatment-experienced, the presence of SMV RASs was associated with a 5% and 3% reduction in SVR12, respectively. Combining host and viral characteristics, only 69% of GT1a, treatment-experienced cirrhotic patients with baseline SMV RASs achieved SVR12, compared to 79% SVR12 rates in those without baseline SMV RASs.

In the SMV/SOF + RBV cohort (n = 58), baseline SMV RASs was associated with 4% lower SVR12 rates (86% vs 90%) (Fig. 5B). The presence of SMV RASs in GT1a was associated with 9% lower SVR12 rates (86% vs 95%; p = 0.38), but SMV RASs had no impact in GT1b. Similar to the SMV/SOF cohort, baseline SMV RASs was associated with 6% lower SVR12 rates in patients who received ≤98 days of therapy, but had no impact on SVR12 rates in patients treated for >98 days (Fig. 5B). Baseline SMV RASs had a larger impact on SVR12 rates in GT1a, treatment-experienced cirrhotics (85% vs 100%; p = 0.49), although the number of GT1a, treatment-experienced cirrhotic patients was small. In both SMV/SOF and SMV/SOF + RBV cohorts, Q80K/R substitutions had a small but detectable impact on SVR12 (Fig. 5C), especially in GT1a. In addition, the presence of R155K or D168E substitutions were associated with lower SVR12 rates, although the number of patients who harbored these RASs was small. Baseline NS5A RASs had no impact on SVR12 rates in SMV/SOF or SMV/SOF + RBV cohorts (Supplmentary Figure S4).

Interestingly, a pooled analysis of all patients from the 4 cohorts revealed that 100% of patients (19/19) with baseline RASs treated for more than 98 days achieved SVR12, whereas 81/93 patients (87%) with baseline RASs treated for ≤98 days achieved SVR12 (p = 0.21; Supplementary Figure S3). This 19-patient cohort was predominantly GT1a (74%, 14/19), treatment-experienced (84%, 16/19), and was mostly DAA-naïve (only 3 had prior exposure to telaprevir), and a large proportion of patients had cirrhosis (74%, 14/19). The median duration of therapy was 168 days (range: 99–176). The clinical characteristic of these 19 patients is shown in Supplementary Table S5. In contrast, in patients without baseline RASs, 95% (77/81) of patients treated for >98 days achieved SVR12 and 93% (259/278) treated for ≤98 days achieved SVR12.

### Abundance and linkage of specific RASs

In an exploratory analysis, we employed a novel quantitative single-variant sequencing (SVS) assay^16^ to more accurately quantify the proportions and linkage of RASs in selected baseline samples. Since Y93 RASs confer high level resistance to NS5A DAAs, we first examine all 7 patients in the LDV/SOF cohort (n = 193) with known baseline Y93H RASs using SVS.

**Figure 6:**
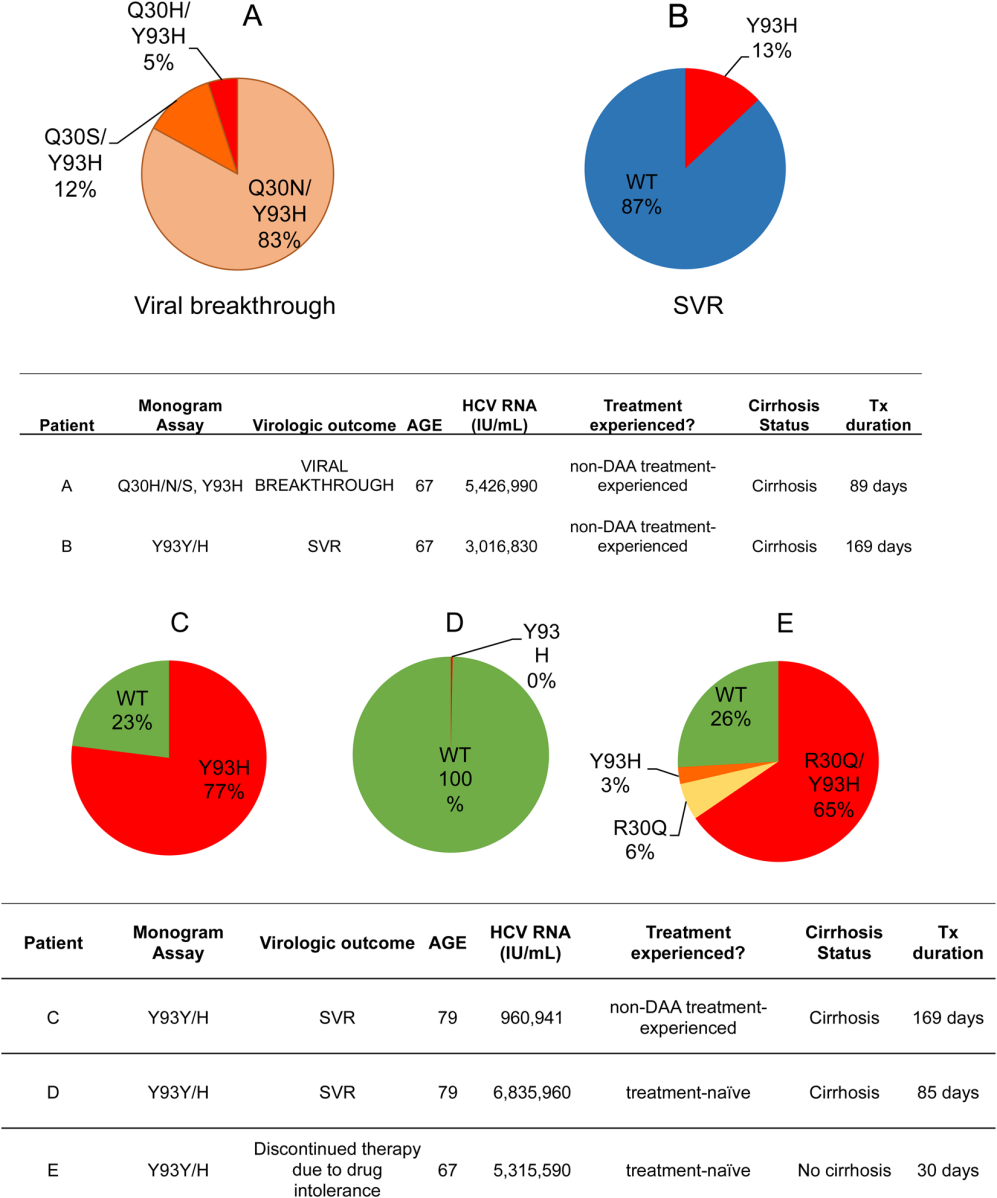
Viral quasispecies composition of (**A**,**B**) GT1a and (**C**–**E**) GT1b patients with baseline Y93H RASs. The relative proportions of RASs in the viral quasispecies are shown in pie charts. Clinical characteristics are shown in the table below.

Of these 7 patients, 2 of 3 GT1a and 2 of 4 GT1b patients had baseline samples available for SVS analysis. Of the 3 GT1a patients, one failed to achieve SVR12 and harbored baseline Q30H/N/S + Y93H substitutions (based on commercial assay) prior to LDV/SOF therapy. Subsequent analysis using quantitative SVS revealed that 100% of the baseline virus had dual RASs, with 83% Q30N/Y93H, 12% Q30S/Y93H, and 5% Q30H/Y93H (Fig. 6A). Of the remaining 2 patients (both achieved SVR12), one had a single RAS (Y93Y/N by commercial assay) and harbored 13% Y93N (Fig. 6B), and the second patient had two RASs (M28M/V + Y93Y/C), but no remaining samples from this patient were available for further SVS analysis.

All 4 GT1b patients with baseline Y93 RASs achieved SVR12 and all 4 had Y93Y/H as determined by commercial resistance assay. One patient had Y93H variant in 77% of the baseline viral population (Fig. 6C), and a second patient had only 0.3% of Y93H (Fig. 6D). Two other patients had no baseline samples available for further analysis. Interestingly, while both samples (Fig. 6C and D) were determined to have Y93Y/H by commercial assay, their viral quasispecies compositions were drastically different (Fig. 6C and D). In an exploratory analysis, we sequenced an additional sample from a patient with known baseline Y93Y/H substitutions who discontinued LDV/SOF early due to drug intolerance (Fig. 1; this patient was excluded from the efficacy analysis). Quantitative SVS revealed that 65.4% of the baseline virus had dual R30Q/Y93H substitutions that were linked, R30Q and Y93H single substitutions were present at 5.9% and 2.7%, respectively, and 25.9% of the baseline population had no major NS5A RASs (Fig. 6E).

To further characterize baseline virus in patients with virologic failure, we analyzed all remaining patients who failed LDV/SOF and had samples available for quantitative SVS and linkage analysis (5 GT1a and 2 GT1b). Of these 7 patients, only one had baseline RASs that were detectable by quantitative SVS (threshold of detection ~0.1–0.5%). This patient had a GT1b virus, characterized by a dual P58S/A92T RASs at a frequency of 2.9%, P58S single substitution at 95%, and A92T single substitution at 1.5%. The remaining 6 patients had no NS5A RASs detectable by either the Monogram Assay or SVS.

**Figure 7:**
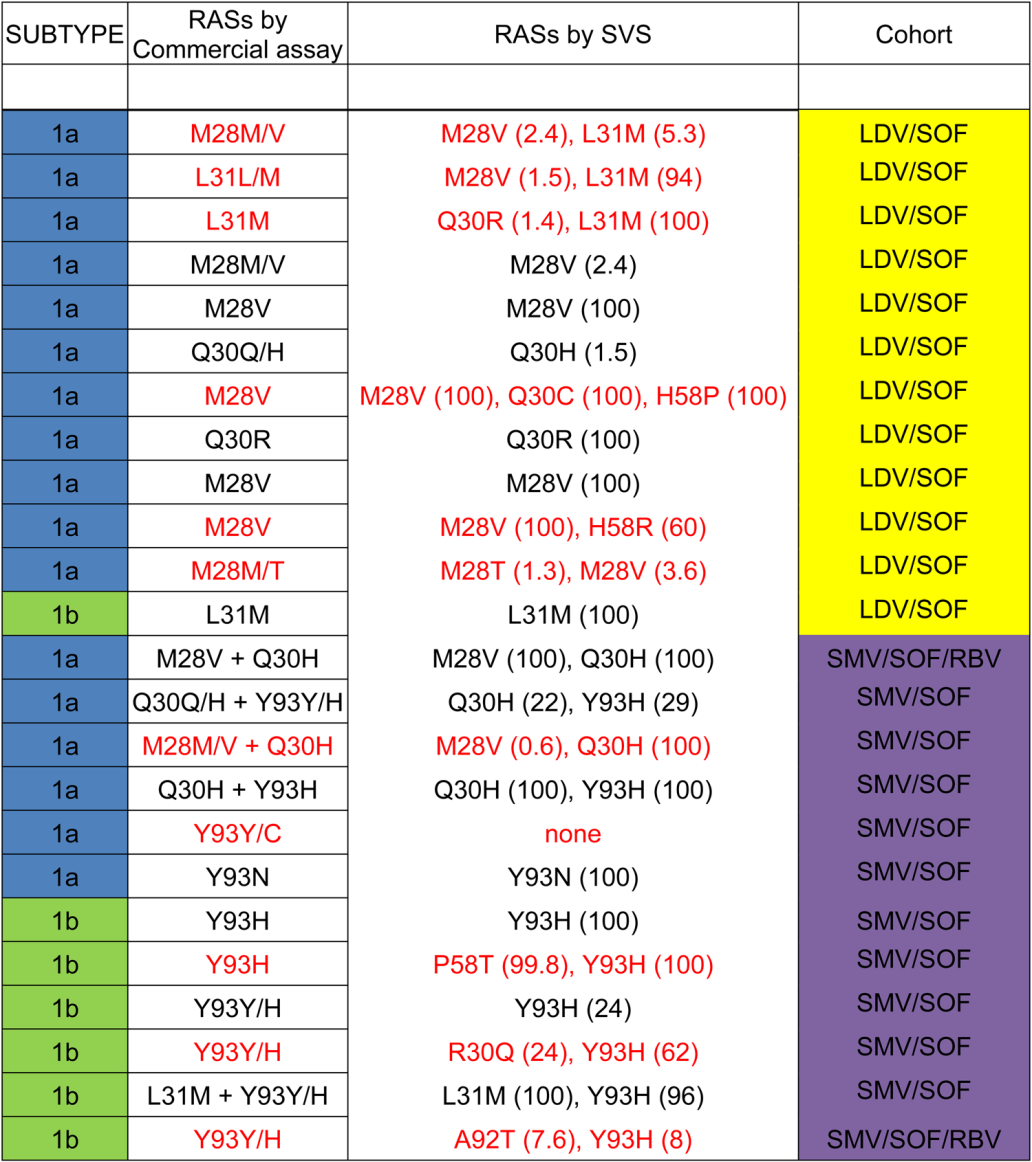
Comparison of RASs dataset between Monogram assay and quantitative SVS in patients with baseline NS5A RASs. A broader list of RASs and substitutions was considered for the SVS assay. Discordant results are shown in red.

To further explore RASs linkage, we sequenced additional 12 subjects in the LDV/SOF cohort and 12 subjects in the SMV/SOF ± RBV cohort who had known NS5A RASs identified by the Monogram assay. All 24 subjects had achieved SVR12. Interestingly, while data between Monogram assay and the quantitative SVS assay were concordant for most samples, differences in RASs were detectable in some samples, and the proportions of viral quasispecies and RASs linkage were identifiable using the SVS analysis (Fig. 7).

Finally, we examined baseline RASs in 5 GT1a patients who relapsed after SMV/SOF treatment and had baseline samples available. Two of the 5 GT1a subjects harbored Q80K substitutions that were detectable by both the Monogram assay and the SVS assay (both had a frequency of 100%). Of the 3 remaining patients with baseline NS3 RASs identified by Monogram assay, one harbored a baseline virus with dual R155K/D168E RASs at 7%, and R155K and D168E single substitutions at 8% and 90%, respectively. A second patient had S122G RAS at 1.5%, and a third patient had no baseline NS3 RASs (Supplementary Table S2).

## Discussion

High replication rate of HCV coupled with error-prone HCV RNA-dependent RNA polymerase leads to the quasispecies nature of HCV populations. Although wild-type viruses are fit and generally outcompete viruses with pre-existing RASs, some variants (e.g. NS5A RASs) may pre-exist and circulate at high frequency within the viral swarm that may impact response to DAA therapy. A number of clinical trials have reported the impact of baseline RASs on outcomes of DAAs therapy. However, the prevalence and impact of baseline RASs in postmarketing, diverse, real-world cohorts of patients treated outside of clinical trials remains unclear. Leveraging the data and specimen repository of the prospective, multi-center, observational cohort study of HCV-TARGET, we report the prevalence and impact of baseline NS3, NS5A, and NS5B RASs in real-world clinical practices in patients treated with ledipasvir/sofosbuvir or simeprevir/sofosbuvir, with or without ribavirin. We found that the overall prevalence of baseline RASs in clinical practice is generally comparable to data from clinical trials. SVR rates across different patient subgroups did not differ significantly according to baseline RASs, but lower SVR12 rates were observed in certain patient subgroups. Interestingly, all 19 patients with baseline RASs who were treated for more than 98 days with one of the 4 regimens analyzed in this study achieved SVR12. In selected patients, linkage between RASs and their proportions could be determined using quantitative single-variant sequencing analysis.

We note that the prevalence of RASs in a given population is dependent on the sequencing methods used for detection, and thus the comparison of prevalence data between studies will require knowledge of sequencing and analysis methods used. For example, minor variants constituting less than 10–20% of the viral population may not be detectable by Sanger population sequencing^18,19^, but RASs as low as 1% of the quasispecies could be detected using next-generation sequencing (NGS)^16,20^, although the clinical relevance of these low frequency variants remains to be defined. To determine the prevalence of baseline RASs and their impact on treatment response in patients in clinical practice, we chose the LabCorp/monogram resistance assay that is widely used in clinical practice. This assay uses Illumina MiSeq deep sequencing with a 10% cutoff to define the presence or absence of a specific RAS.

Not surprisingly, the prevalence of RASs varied according to gene targets and amino acid position. The overall prevalence of NS3, NS5A, NS5B RASs (determined at 10% threshold) was 45%, 13%, and 8%, respectively, which are comparable to previous literature. For NS3, the prevalence of baseline RASs varies widely in the literature, ranging from 5.5% to 46%^21–23^. This wide variation largely depends on whether Q80 RASs are included in the analysis. We found that baseline Q80K RAS was common (45%) in GT1a patients, but uncommon (3%) in GT1b patients. However, other clinically important NS3 RASs often selected following virologic failure of PI-containing regimens, including R155K, A156S/T/V, and D168 substitutions, were uncommon (<3%) in real world practices. For NS5A, baseline RASs at positions 28, 30, 31, 58, 93 were found in 13% of our patient population (12% in GT1a and 17% in GT1b), which is in agreement with the range of 5–15% in published reports^18,24^. For GT1a, M28T/V was most common (6%), comparable to published data of 4–8%^21,23^, but Y93C/H/N, a RAS that confers high-level resistance to NS5A inhibitors^25^, was uncommon (3%). In contrast, Y93C/H/N was common in GT1b (11%), followed by L31M/I (7%). For NS5B, baseline sofosbuvir RASs were uncommon (4%), and S282T, L320 or V321 substitutions were not detected in any of the 492 baseline samples. On the other hand, dasabuvir RASs were prevalent (22%) in GT1b but not in GT1a patients, due primarily to the high prevalence of C316N (8%) and S556G (14%) in GT1b viruses which is higher than previous reports^26^, although C316N may be a polymorphism commonly seen in GT1b in some parts of the world^27,28^. Within different patient subgroups, the prevalence of RASs was generally comparable between cirrhosis and non-cirrhosis, treatment-naïve (TN) and treatment-experienced (TE), and irrespective of liver transplantation status. Interestingly, 10% of patients in real world practices harbored RASs in 2 or more drug classes, with NS3/NS5A dual-class RASs being the most common, followed by NS3/NS5B RASs then NS5A/NS5B RASs. The very small number of patients with RASs in 2 or more classes in different treatment cohorts precluded a detailed analysis of their impact on treatment outcome.

While the presence of baseline RASs has impacted viral outcomes in small numbers of patients in controlled clinical trials, the extent to which they impact efficacy of DAAs in the general population and in certain sub-populations in routine clinical practice remains unclear. Simeprevir (SMV) plus sofosbuvir (SOF) was the first interferon-free, all oral regimen that became available in late 2013 and is well tolerated with SVR rates of >90% in most patient subpopulations. However, the Q80K polymorphism, common in GT1a (45% prevalence in the present study), confers 7 to 10-fold increase in EC50 to simeprevir^29^. In OPTIMIST-1 and -2 registration trials of patients treated with 8 or 12 weeks of simeprevir plus sofosbuvir, the presence of baseline Q80K had a significant impact on SVR12 rates of patients with GT1a cirrhosis (25/34 or 74%), compared to other patient subgroups^30^. Consistent with data from registration trials, the presence of baseline Q80K or SMV RASs in the HCV-TARGET cohort had a small impact on SVR12 rates in each individual patient subgroup (i.e. cirrhosis, TE, or GT1a). However, when these unfavorable host and viral factors are present simultaneously (i.e. the GT1a, treatment-experienced cirrhotic subgroup) in the SMV/SOF cohort, those with baseline SMV RASs had lower SVR12 rates compared to those without baseline RASs (69% vs 79%). Similar trends were observed in patients treated with simeprevir plus sofosbuvir plus ribavirin (85% vs 100%, respectively).

Ledipasvir (LDV) plus sofosbuvir (SOF) is a well tolerated, all oral regimen with SVR rates in the real world over 90–95%^31^. In ION-1 and −3 registration trials, SVR12 rates were over 97% across all arms with no significant difference in SVR based on length of treatment, use of ribavirin, cirrhosis status, or HCV GT1 subtype^6–8^. In the present study, the presence of LDV RASs (at positions 28, 30, 31, 58, 93) was associated with a small reduction of 3% in SVR12 rates in the LDV/SOF cohort (a range of 1–7% reduction in SVR12 rates across different patient subgroups including cirrhosis status, GT1 subtype, and TN vs TE). Interestingly, although Y93 RASs were rare (4%), one of the three GT1a patients with baseline Y93H failed to achieve SVR12, which may be due in part to its more complex quasispecies populations (Fig. 6A) that may confer a higher level of resistance to NS5A inhibitors.

Current commercial HCV resistance assays rely on either population sequencing or next generation sequencing (NGS). Population sequencing reports the sequence of the predominant circulating HCV variants, which is insensitive in detecting minority variants present in less than 10–15% of viral quasispecies population. Although deep sequencing is more sensitive, the conventional sequencing approach suffers from technical artifacts, which could arise from amplification errors and bias associated with reverse transcription and PCR, resampling errors from low copy number of viral templates, and sequencing errors^16,32,33^. Since the commerical lab did not provide quantitative data and instead reported only the population sequence using a pre-defined variant reporting threshold (10% cutoff in the present study), data between commercial labs and qSVS could not be compared quantitatively (Fig. 7). A 10–15% threshold is widely used in research settings and a cut-off of 15% was recommended by the recent EASL guideline^34^. Our study used a pre-defined 10% cut-off from the commercially available Monogram assay, which may have resulted in a slightly higher overall prevalence of RASs compared to a 15% cut-off. However, the percent cut-off that is clinically relevant for each specific regimen is currently unknown. In addition, deep sequencing assays are generally performed without correcting for potential technical errors as described above, and the information regarding linkage of RASs is not reported in commercial assays. Our data suggest that mutation linkage may be clinically relevant and impact treatment response. For example, viruses with NS5A dual Q30H/Y93H substitutions may confer higher level of resistance against NS5A inhibitors compared to viruses with Q30H or Y93H single substitutions.

To our knowledge, the impact of relative abundance and linkage of RASs on treatment response has not been previously investigated. Using a recently developed quantitative single-variant sequencing (qSVS) assay^16^ that potentially overcomes technical artifacts as described above, we conducted a secondary exploratory analysis of selected baseline samples to more accurately quantify the proportions and determine the linkage of variants within viral populations. Of the 7 patients (3 GT1a and 4 GT1b) who had baseline Y93 RASs detected by the Monogram assay, 1 of 3 GT1a patients failed to achieve SVR12, but all 4 GT1b patients achieved SVR12. Interestingly, analysis of baseline RASs by SVS revealed that the one GT1a patient who failed to achieve SVR12 harbored a virus with 100% dual RASs (Q30 linked to Y93H). In contrast, one of the 2 GT1a patients who achieved SVR12 harbored Y93N RAS in only 13% of the viral population (the third patient had no samples remaining for SVS analysis). Although Y93N is known to confer higher level resistance to NS5A DAAs compared to Y93H (>10-fold higher), the lower frequency of Y93N (13%) in the viral quasispecies population may have allowed viral suppression and SVR12 by LDV/SOF combination therapy. However, these data should be interpreted with caution given the low frequency of Y93 RASs in the real world cohort and the small sample size with baseline RASs. Nonetheless, these results raise the possibility that a high proportion of highly resistant virus in the HCV quasispecies population may contribute to treatment failure, which could also explain the generally lower SVR12 rates during re-treatment of patients with detectable baseline NS5A RASs.

There are several weaknesses in this study. First, since this a real-world cohort and not a randomized control trial, patients received one of four possible regimens with varying treatment durations. Given our sample size, the low frequency of baseline Y93 RASs in the general population made it difficult to fully assess their impact on outcome. Secondly, it is possible that patients receiving ribavirin were considered by the treating providers to have unfavorable baseline characteristics which could have led to a bias for baseline RASs in the ribavirin-containing cohorts. Indeed, a higher proportion of patients with cirrhosis or prior DAA exposure was observed in the ribavirin-containing cohorts (Table 1). While the knowledge of baseline NS3 RASs (e.g. Q80K) may have influenced some treating physicians to add ribavirin, Q80K polymorphism data was available in only 18% of patients in SMV-containing cohorts. The distribution of RASs reported in this study may also be different than the overall population with HCV, as samples from only a subset of HCV-TARGET patients (~5%) were studied and the age range of our cohort suggests an older population with likely more advanced liver disease. Finally, we could not fully address the impact of RASs in DAA-experienced patients because of the small sample size (only 63 patients or 12.8% of total), and as several newer regimens are now available, the importance of SMV-containing regimens will diminish.

To our knowledge, this is the first study that reports the prevalence and impact of baseline RASs in patients treated with DAAs in routine clinical practice. Our results demonstrate that the overall prevalence of RASs was generally comparable to data in registration trials and between different patient subgroups in clinical practice. The presence of baseline LDV or SMV RASs was associated with a small but detectable reduction in SVR12 rates in select patient subpopulations, and the quasispecies population of the baseline virus may contribute in part to treatment failure. Importantly, all 19 patients with baseline RASs treated for more than 98 days achieved SVR12, whereas 87% treated for ≤98 days achieved SVR12, suggesting that longer treatment duration may overcome the impact of baseline RASs on SVR12 rates in DAA-naïve patients. These data provide additional support for the clinical practice of extending treatment course to overcome the effects of unfavorable characteristics including baseline RASs, particularly in settings where SMV/SOF and LDV/SOF regimens are still used and where RAS testing may not be readily available. Emerging research is now focusing on specific patient subpopulations especially DAA-experienced patients that may be most impacted by the presence of different combinations of treatment emergent RASs. However, the role of RASs may be diminished with a broader use of more recently approved DAAs^9^. As SVS analysis has the potential to provide detailed characterization of HCV quasispecies populations including RASs linkage, this approach may offer additional insights during re-treatment of DAA-experienced patients in future studies.

## Electronic supplementary material

Supplementary Information
