# Supplementary material for: Prevalence and impact of baseline resistance-associated substitutions on the efficacy of ledipasvir/sofosbuvir or simeprevir/sofosbuvir against GT1 HCV infection
Source: Sci Rep. 2018 Feb 16;8:3199. doi: 10.1038/s41598-018-21303-2 (PMC5816647; doi:10.1038/s41598-018-21303-2)
Supplement: Supplementary file 1 — Supplementary Information [file 41598_2018_21303_MOESM1_ESM.docx]

**Supplementary Information**

**Prevalence and impact of baseline resistance-associated substitutions on the efficacy of ledipasvir/sofosbuvir or simeprevir/sofosbuvir against GT1 HCV infection**

Gary P. Wang^* 1, 2^, Norah Terrault^3^, Jacqueline D. Reeves^4^, Lin Liu^* 1,^, Eric Li^* 1,^, Lisa Zhao^* 1,^, Joseph K. Lim^5^, Giuseppe Morelli^1^, Alexander Kuo^6^, Josh Levitsky^7^, Kenneth E. Sherman^8^, Lynn M. Frazier^9^, Ananthakrishnan Ramani^10^, Joy Peter^1^, Lucy Akuskevich^11^, Michael W. Fried^11^, and David R. Nelson^1^

^1^University of Florida, Gainesville,

^2^North Florida/South Georgia Veterans Health System, Gainesville,

^3^University of California, San Francisco, San Francisco,

^4^Monogram Biosciences, South San Francisco,

^5^Yale University School of Medicine, New Haven,

^6^Virginia Mason Medical Center, Seattle,

^7^Northwestern University Feinberg School of Medicine, Chicago,

^8^University of Cincinnati, Cincinnati,

^9^Liver Wellness Center, Little Rock,

^10^Columbia Memorial Hospital (Mountainview Medical Center), Hudson,

^11^University of North Carolina, Chapel Hill, United States

**Supplementary Table S1.** Positions of RASs Analyzed

**Supplementary Table S2.** Comparison of RASs dataset between Monogram assay and quantitative SVS in GT1a patients who relapsed after SMV/SOF therapy

| **SUBTYPE** | **RASs by Monogram assay** | **RASs by SVS** | **AGE** | **HCV RNA (IU/mL)** | **Treatment experienced?** | **Cirrhosis Status** | **Tx duration** |
| --- | --- | --- | --- | --- | --- | --- | --- |
| 1a | Q80K | Q80K (100) | 57 | 410 | treatment-naïve | Cirrhosis | 85 days |
| 1a | none | S122G (1.5) | 55 | 722666 | non-DAA treatment-experienced | Cirrhosis | 84 days |
| 1a | none | none | 60 | 82500 | non-DAA treatment-experienced | Cirrhosis | 85 days |
| 1a | none | R155K/D168E (7), R155K (8), D168E (90) | 64 | 39000000 | treatment-naïve | Cirrhosis | 84 days |
| 1a | Q80K | Q80K (100) | 67 | 51200000 | treatment-naïve | Cirrhosis | 84 days |

**Supplementary Figure S3. Effect of treatment duration based on the presence or absence of baseline RASs.** Top: Proportions of patients pooled from all 4 cohorts who were treated with ≤ 98 days of therapy are shown in red. Patients treated with >98 days of therapy are shown in blue. Baseline RASs are defined as RASs relevant for the treatment regimen that the patients received (e.g. LDV and SOF-associated RASs were considered for patients treated with LDV/SOF). Bottom: Proportions of patients treated with ≤ 98 days of therapy or > 98 days of therapy are shown in red and blue bars, respectively. LDV RASs analyzed were M28A/T/V, Q30E/H/L/R, L31I/M/V, H58D, Y93C/H/N/S. SMV RASs analyzed were Q80K/R, S122A/G/I/R/T, R155K/Q, D168A/E/F/H/I/T/V/Y, IV170/T. SOF RAS analyzed were L159F, S282R/T, C316F/H/N, L320F/I/V, V321A/I.

**Supplementary Figure S4. (A) Impact of baseline NS3 RASs on SVR12 in LDV/SOF ± RBV cohorts, and (B) Impact of baseline NS5A RASs on SVR12 in SMV/SOF ± RBV cohorts.** The proportions of patients with or without indicated baseline RASs who achieved SVR12 are shown in red and blue bars, respectively. P > 0.05 in all four comparisons.

**Supplementary Table S5.** Demographics and baseline characteristics of 19 subjects with baseline RASs who were treated for more than 98 days and achieved SVR12.
